# RNA-sequencing analysis reveals the potential contribution of lncRNAs in palmitic acid-induced insulin resistance of skeletal muscle cells

**DOI:** 10.1042/BSR20192523

**Published:** 2020-01-02

**Authors:** Mei Han, Lianghui You, Yanting Wu, Nan Gu, Yan Wang, Xiaodan Feng, Lanlan Xiang, Yajun Chen, Yu Zeng, Tianying Zhong

**Affiliations:** 1 Women’s Hospital of Nanjing Medical University (Nanjing Maternity and Child Health Care Hospital), Nanjing 210004, China; 2 Affiliated Maternity and Child Health Care Hospital of Nantong University, NanTong 226006, China

**Keywords:** Insulin resistance, LncRNA, Palmitic acid, Skeletal muscle

## Abstract

Insulin resistance (IR) has been considered as the common pathological basis and developmental driving force for most metabolic diseases. Long noncoding RNAs (lncRNAs) have emerged as pivotal regulators in modulation of glucose and lipid metabolism. However, the comprehensive profile of lncRNAs in skeletal muscle cells under the insulin resistant status and the possible biological effects of them were not fully studied. In this research, using C2C12 myotubes as cell models *in vitro*, deep RNA-sequencing was performed to profile lncRNAs and mRNAs between palmitic acid-induced IR C2C12 myotubes and control ones. The results revealed that a total of 144 lncRNAs including 70 up-regulated and 74 down-regulated (|fold change| > 2, *q* < 0.05) were significantly differentially expressed in palmitic acid-induced insulin resistant cells. In addition, functional annotation analysis based on the Gene Ontology (GO) and Kyoto encyclopedia of genes and genomes (KEGG) databases revealed that the target genes of the differentially expressed lncRNAs were significantly enriched in fatty acid oxidation, lipid oxidation, PPAR signaling pathway, and insulin signaling pathway. Moreover, Via qPCR, most of selected lncRNAs in myotubes and db/db mice skeletal muscle showed the consistent expression trends with RNA-sequencing. Co-expression analysis also explicated the key lncRNA–mRNA interactions and pointed out a potential regulatory network of candidate lncRNA ENSMUST00000160839. In conclusion, the present study extended the skeletal muscle lncRNA database and provided novel potential regulators for future genetic and molecular studies on insulin resistance, which is helpful for prevention and treatment of the related metabolic diseases.

## Introduction

Insulin resistance (IR) is considered as the common pathological basis and progressive driving force for several metabolic diseases, including obesity, Type 2 diabetes mellitus (T2DM), hyperlipidemia, hypertension, and coronary heart disease [1–3]. To maintain whole-body metabolic homeostasis, intensive interest has been focused on exploring the molecular mechanisms underlying the development of IR.

The main feature of IR is impaired of physiological responses to normal insulin concentrations in peripheral target tissues including muscle, liver and adipose tissues. Therefore, IR is characterized by the defective of insulin sensitivity in related tissues, which leads to a disturbance in glucose and lipid metabolism. Skeletal muscle, accounting for about 40% of the body mass, is both a locomotive organ and an important organ for 80% of the body’s glucose uptake, which is used to maintain metabolic balance [4–6]. Evidence has confirmed the importance of skeletal muscle responsible for modulating peripheral insulin sensitivity and whole-body metabolism. For example, perturbed heterogeneous nuclear ribonucleoprotein A1-induced turbulences in glucose homeostasis in the skeletal muscle could accelerate systemic IR under both basal and high-fat induced conditions [7]. Conversely, activating mTOR signaling, improved insulin sensitivity in the skeletal muscle, which could enhance whole-body metabolism via weight loss and an increased fatty acid oxidation capacity in the adipose tissue and liver [8,9]. Studies have shown that among the widely recognized mechanisms underlying IR in the skeletal muscle, quite a few studies displayed that accumulation of ‘toxic’ lipid metabolites was considered as an important contributor to IR in skeletal muscle [10–14]. Lipid-induced IR has been associated with different cellular events in skeletal muscle cells, including impaired insulin signaling transduction of IRS1–PI3K [15], activation of inflammatory and pro-inflammatory cytokines [16], abnormally accumulation of ceramides [17], and mitochondrial dysfunction [18]. Therefore, the factors or the mechanisms underlying IR in the skeletal muscle, which are induced by lipid overload, must be explored.

Long noncoding RNAs (lncRNAs), transcribed by RNA polymerase II, is a kind of RNA molecules with lengths of more than 200 nucleotides and without evident protein-coding capacity. The present researches have demonstrated that most lncRNAs regulate gene expression at epigenetic, transcriptional and/or post-transcriptional levels [19,20]. Specific location and patterns of lncRNAs coordinate embryonic development and cell differentiation, while abnormal location and patterns of lncRNAs could contribute to diseases development [21,22], especially in several metabolic diseases [23]. Notably, LncRNAs participate in glycolipid metabolism dysfunction in pancreatic beta cells [24], liver [25] and adipocytes [26]. Most studies that have explored the effects of lncRNAs on skeletal muscle function, the majority regarded the skeletal muscle as a locomotive organ and focused their attention on skeletal myogenesis and muscle atrophy [27]. It was widely reported that lncRNAs, such as MUNC [28] and Linc-RAM [29], were reported to be closely related to myogenesis and post-injury regeneration in skeletal muscle. However, the link of lncRNAs to insulin sensitivity in the skeletal muscle is just beginning to be revealed. Thus, the repertoires and functional identifications of lncRNAs in insulin resistant muscle should be deserved to be described.

The palmitic acid (PA) induced skeletal muscle IR model is widely approved and used as an ideal cell model *in vitro* for exploring potential regulatory factors as well as the mechanisms underlying IR in skeletal muscle. The present study determined the lncRNAs profiles of the C2C12 myotubes between PA-treated and control samples. A total of 144 lncRNAs (70 up-regulated and 74 down-regulated; |fold change| > 2, *q* < 0.05) were significantly differentially expressed in the PA-treated myotubes. The potential functions of the differentially expressed lncRNAs were identified by cluster analyzing protein-coding genes with gene ontology (GO) enrichment and Kyoto Encyclopedia of Genes and Genomes (KEGG) pathway analysis. qRT-PCR method was used to detect differentially expressed lncRNAs in RNA-sequencing data. About 5 of 12 lncRNAs were consistent in the PA-treated C2C12 myotubes and IR mouse models (*db/db* mice). Coexpression analysis between differentially expressed mRNAs and candidate lncRNAs demonstrated the key lncRNA–mRNA interactions and suggested a potential regulatory network of candidate lncRNA ENSMUST00000160839. Our results help to expand the data on lncRNAs in the skeletal muscle cells and identified IR-related regulators, thus providing a potential strategy for preventing and treating T2DM and the related metabolic diseases.

## Materials and methods

### Cell culture and treatments

C2C12 myoblast cells were purchased by Stem Cell Bank, Chinese Academy of Sciences (Shanghai, China). C2C12 myoblast cells were cultured in Dulbecco’s modified Eagle’s medium (DMEM) (Gibco, Grand Island, CA, U.S.A.) supplemented with 10% fetal bovine serum (Gibco, Scoresby VIC, Australia) and 1% penicillin/streptomycin (Wisent, Nanjing, China) in a humidified 5% CO_2_ atmosphere at 37°C. Two days after reaching 80–90% confluence, the cells were differentiated with medium containing DMEM (Gibco), 2% horse serum (Gibco, Auckland, New Zealand) and 1% penicillin/streptomycin (Wisent). The differentiation medium was changed every 2 days. After 4 days, the differentiated myotubes were starved with serum-free medium for 3–5 h before treatment with PA (Sigma-Aldrich, St.Louis, MO, U.S.A.). C2C12 myotubes were exposed to 2% bovine serum albumin/DMEM medium for 24 h with or without 0.75 mM PA according to our previous study [30]. Then two groups of myotubes were then collected with TRIzol reagent (Invitrogen, Carlsbad, CA, U.S.A.) and stored at −80°C.

### Animal experiments

The Ethical Committee of Nanjing Medical University approved all animal procedures. All animal experimental protocols were in compliance with the relevant guidelines and ordinances of the Institutional Animal Care and Use Committee (IACUC-1812053). Twelve-week-old male C57BLKS/J *db/db* mice (*n* = 8) and age-matched wild-type (WT) controls (*n* = 8) were purchased from the Model Animal Research Center of Nanjing Medical University. All mice were maintained under the standard conditions with a 12-h:12-h light–dark cycle, at 22 ± 2°C, and 40 ± 10% humidity. The skeletal muscles were isolated and immediately frozen with liquid nitrogen and stored at −80°C until RNA extraction. The frozen skeletal muscle was homogenated with a homogenizer (IKA, Staufen, Germany) and extracted in TRIzol reagent (Invitrogen).

### RNA isolation, library preparation, and sequencing

Total RNA was isolated from the C2C12 myotubes using an RNeasy mini kit (Qiagen, Hilden, Germany) following the manufacturer’s protocols. The TruSeq® Stranded Total RNA Sample Preparation kit (Illumina, San Diego, CA, U.S.A.) was used to prepare the strand specific libraries according to the manufacturer’s instructions. After removing ribosomal RNA from the total RNA by using a VAHTS Total RNA-Seq (H/M/R) Library PrepKit for Illumina (Vazyme, Nanjing, China), the mRNA was fragmented into small pieces using divalent cations at 94°C for 8 min. The cleaved RNA fragments were copied into first-strand cDNA, then into second strand cDNA by using reverse transcriptase (SuperScript II Reverse Transcriptase, Thermo Fisher Scientific, Waltham, MA, U.S.A.), random primers, DNA polymerase I and RNase H, respectively. With these cDNA fragments processed through an end repair process, the addition of a single ‘A’ base, and ligation of the adaptors, the products were then purified by using Agencourt® AMPure XP Beads (Beckman, Coulter, Germany) and enriched with PCR by using a Qubit™ dsDNA HS Assay Kit (Thermo Fisher Scientific) to create the final cDNA library. Purified libraries were quantified with a Qubit® 2.0 Fluorometer (Life Technologies, Carlsbad, CA, U.S.A.) and validated using an Agilent 2100 bioanalyzer (Agilent Technologies, Santa Clara, CA, U.S.A.) to confirm the insert size and calculate the mole concentration. Cluster was generated by cBot with the library diluted to 10 pM and then sequenced on the Illumina HiSeq X-ten (Illumina). The library was constructed and sequencing was performed on 6 samples (3 WT and 3 PA-treated C2C12 myotubes) at Shanghai Biotechnology Corporation.

### Differentially expressed lncRNAs and mRNAs analysis

Hisat2 (version: 2.0.4) [31] was used to map the clean reads to the GRCm38 reference genome after filtering out ribosomal RNA reads, sequencing adapters, short-fragment reads and other low-quality reads to preprocess the sequencing raw reads; Hisat2 (version: 2.0.4) [31] was used to map the clean reads to the GRCm38 reference genome. Stringtie (version: 1.3.0) [32,33] was run with a reference annotation to generate Fragments per Kilobase Million (FPKM) values for known gene models after genome mapping. EdgeR [34] was used to identify differentially expressed lncRNAs and mRNAs. The false discovery rate (FDR) was used to set the *P*-value significance threshold in multiple tests, while the fold-changes were also estimated according to the FPKM in each sample. The differentially expressed lncRNAs and mRNAs were selected with the following filter criteria: |fold change| ≥ 2, and FDR ≤0.05.

### GO and KEGG enrichment analysis

The predicted potential lncRNAs were integrated with the differentially expressed mRNAs in the sequencing. GO terms and KEGG pathways were assigned to determine the functions and pathways of the differentially expressed mRNAs. We mapped differentially expressed mRNAs to GO terms in the database (http://www.geneontology.org) and then calculated gene numbers for each term. In addition, the KEGG pathway analysis (http://www.kegg.jp/) was used to confirm the enriched pathways. The threshold of significance was defined by the *P*-value (*P* < 0.05 was considered statistically significant).

### RNA extraction, reverse transcription and real-time PCR analysis

Total RNA was extracted from the cells and tissues using TRIzol reagent (Invitrogen) according to the manufacturer’s instructions. RNA concentration and purity were analyzed using a NanoDrop ND-2000 spectrophotometer (Agilent, Santa Clara, CA, U.S.A.), and the range of the OD260 nm/OD280 nm absorption ratio was controlled between 1.9 and 2.2. A total of 2 μg RNA from each sample was reverse transcribed with a Thermo Scientific™ RevertAid First Strand cDNA Synthesis kit (Thermo Fisher Scientific) using oligo (dT) primers (for mRNA) or random primers (for lncRNAs), respectively. Quantitative PCR (qPCR) was performed using a 384-well Applied Biosystems ViiA system (Life Technologies, Carlsbad, CA, U.S.A.) using the SYBR Green method (Thermo Fisher Scientific) in a 10-μl reaction volume following the manufacturer’s protocols. Primers used for qPCR were designed and confirmed using BLAST (http://www.ncbi.nlm.nih.gov/tools/primer-blast/) to ensure the product specificity. Supplementary Table S1 lists all the qPCR primers. The housekeeping gene, PPIA, was used as an internal reference. LncRNAs and mRNAs expression levels were detected as the *C*_T_ value, calculated using the 2^−ΔΔ*C*^_t_ method, and normalized to PPIA expression as previously reported [30].

### lncRNA–mRNA co-expression network

To identify the interactions among genes, gene co-expression networks were constructed according to the normalized signal intensity of the genes. With no special treatment of the lncRNA expression value, all transcripts data expressed from the same coding gene were preprocessed by using the median gene expression value. We then selected the data of differentially expressed lncRNAs and mRNAs from the dataset. After confirmation of the data that are normally distributed, the network was constructed by calculating the Pearson correlation of each gene pair analyzed and significantly correlated pairs (only lncRNA–mRNA) were chosen. The strongest correlations (≥0.90) were drawn to construct visual representation. Each gene corresponded to a node, and a strong correlation (i.e., either positive or negative) was indicated when two genes were connected by an edge. Cytoscape (National Institute of General Medical Sciences, Boston, MA, U.S.A.) was used to draw the co-expression networks.

### Protein extraction and Western blotting

The C2C12 myotubes protein extracts were subjected to sodium dodecyl sulfate-polyacrylamide gel electrophoresis using GenScript SurePAGE, Bis-Tris, 10 cm × 8 cm gels (GenScript, Nanjing, China). Separated proteins were then transferred to nitrocellulose membranes using tank transfer for 1.5 h at 400 mA in Tris–glycine buffer containing 20% methanol. The membranes were blocked with 5% skim milk for 1 h and incubated overnight with diluted primary antibodies against Akt, p-Akt (ser473), Irs1 and p-Irs1(ser307) (diluted at 1:1000, Cell Signaling Technology, Danvers, MA, U.S.A.), followed by a secondary antibody against rabbit IgG (diluted at 1:4000, Biosharp, Hefei, China). The signal was detected using the FluorChem fluorchemiluminescence system (ProteinSimple, San Francisco, CA, U.S.A.). The optical density (OD) of each band was determined using the Image J v 1.8.0 (National Institute of Mental Health, U.S.A.).

### Statistical analysis

Data were analyzed with GraphPad Prism 7 (GraphPad, Inc. San Diego, CA, U.S.A.), and the results were presented as the mean ± standard error of the mean (SEM). An unpaired two-tailed *t*-test was used to comapre differential gene expression levels. The differences were considered statistically significant at *P* < 0.05.

## Results

### Differential expression analysis of lncRNAs and mRNAs

**Figure 1:**
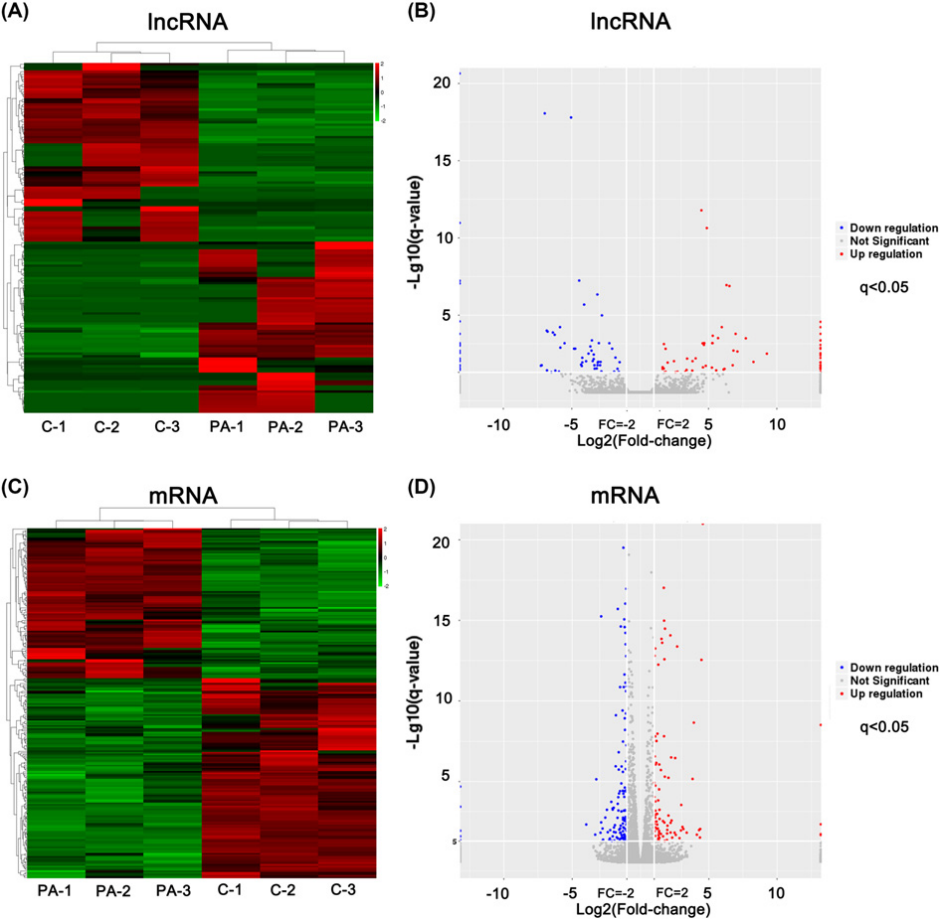
Profile of differentially expressed lncRNAs and mRNAs between PA-treated (PA group) and control C2C12 myotubes (C group) (**A** and **C**) Hierarchical clustering of RNA sequencing indicated lncRNA and mRNA expression profiles in C2C12 myotubes between PA-treated and control samples (three samples per group). Data were expressed as FPKM. Red: relatively high expression; Green: relatively low expression. (**B** and **D**) The scatter plot, a visualization method, was applied for assessing lncRNA and mRNAs expression variation between the two compared groups. The red dots indicated up-regulated RNA and blue dots indicated down-regulated RNA in PA-treated C2C12 myotubes (|fold change| >2, *q* < 0.05).

As commonly reported, palmitic acid (PA), a kind of saturated fatty acid, is well-known to induce insulin resistance in C2C12 myotubes, which is characterized with an impaired Irs1-Akt signal pathway. Using this way, we established insulin-resistant model in C2C12 myotubes *in vitro* (Supplementary Figure S1). We confirmed the model by detecting the insulin-stimulated Irs1 (Ser307) and Akt (Ser473) phosphorylation levels. Insulin could significantly enhance Irs1 (Ser307) and Akt (Ser473) phosphorylation levels in the control groups (C), while the stimulation tendency was weak after PA exposure (Supplementary Figure S1). After validating the insulin-resistant model, we profiled the lncRNAs and mRNAs expressions using RNA sequencing technology. Hierachical clustering presented lncRNAs and mRNAs expressions patterns in C2C12 myotubes in the control and PA-treated groups (Figure 1A,C). A scatter plot, a visualization method, was applied to assess the variations in the lncRNA and mRNA expression between the two compared groups (Figure 1B,D). All screened lncRNAs with a signal, which were altered with |fold change>2| and *q* < 0.05, differed statistically. Clustering and comparision analysis revealed 144 differentially expressed lncRNAs, of which 70 lncRNAs were up-regulated and 74 lncRNAs were down-regulated. Supplementary Table 2 presented a list of differentially expressed lncRNAs.

### Functional enrichment analysis

**Figure 2:**
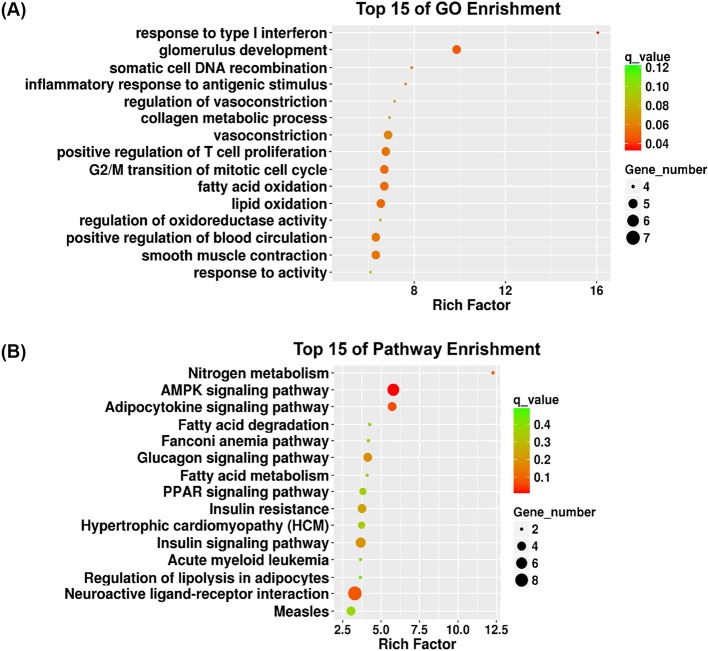
GO analysis and pathway analysis of differentially expressed mRNAs in control and PA-treated C2C12 myotubes (**A**) Top 15 GO terms of differentially expressed mRNAs were listed. (**B**) Top 15 pathway terms of dysregulated mRNAs were listed.

To investigate the possible functions of the differentially expressed lncRNAs in the PA-treated C2C12 myotubes, we performed Gene ontology (GO) analysis which was an international standard classification system of gene function on biological processes (BP). We found that the main GO terms targeted by the dysregulated lncRNAs were “response to type I interferon”, “glomerulus development”, “somatic cell DNA recombination”, “inflammatory response to antigenic stimulus”, “regulation of vasoconstriction”, “collagen metabolic process”, “vasoconstriction”, and “positive regulation of T cell proliferation”. Figure 2A showed the top 15 GO terms; Supplementary Table S3 presented the annotated transcripts involved in these GO terms. Next, we performed KEGG enrichment analysis to predict the potential functions of these lncRNAs. Dysregulated transcripts were enriched in the signaling pathways for “Nitrogen metabolism”, “AMPK signaling pathway”, “Adipocytokine signaling pathway” and “Fatty acid degradation”. Among these biological pathways, the most enriched network was “Nitrogen metabolism” (Fisher’s *P* value = 1.378e-03). Figure 2B listed the top 15 pathways; Supplementary Table S4 presented the annotated transcripts involved in these pathway terms.

### Verification of gene expression profiles using qRT-PCR

**Figure 3:**
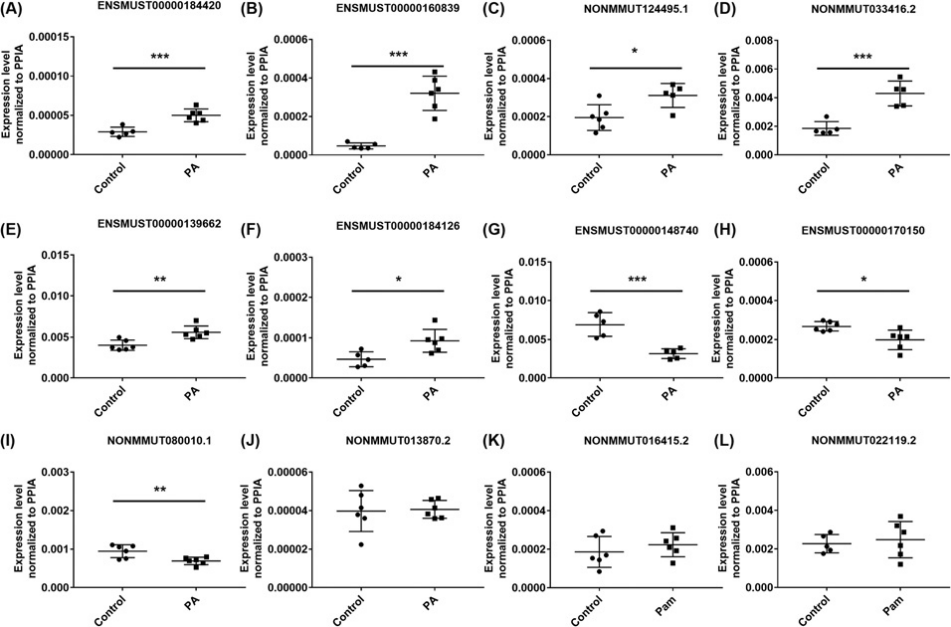
qPCR verification of differentially expressed lncRNAs in PA-treated C2C12 myotubes Differentiated C2C12 myotubes were incubated in either the presence or absence of 0.75 mM concentrations of PA in 2% BSA for 24 h (*n* = 6 per group). The expression levels of 12 differentially expressed lncRNAs were evaluated by qPCR in C2C12 myotubes exposed to PA. (**A–F** and **J–L**): 9 lncRNAs were up-regulated in RNA sequencing and (**G–I**) 3 lncRNAs were down-regulated. 2^−Δ*C*^_T_ was used to show the expression levels of lncRNAs and PPIA expression was used as the internal control for lncRNA expression analysis. Values are the means ± SEM (*n* = 6). *, *P* < 0.05; **, *P* < 0.01; ***, *P* < 0.001.

**Figure 4:**
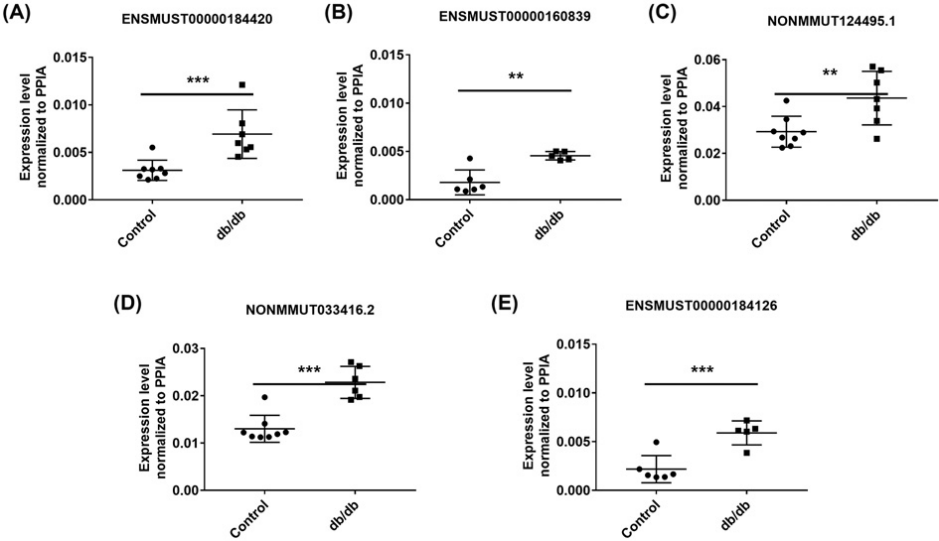
qPCR verification of differentially expressed lncRNAs in skeletal muscle tissues Skeletal muscle tissues were isolated from male C57BLKS/J *db/db* mice at the age of 12 weeks (*n* = 8) and age-matched WT controls (*n* = 8). (**A–E**) The differentially expressed lncRNAs selected from the PA-treated C2C12 cells results were validated in skeletal muscle tissues by qPCR analysis. 2^−Δ*C*^_T_ was used to show the expression levels of lncRNAs and PPIA expression was used as the internal control for lncRNA expression analysis. Values are the means ± SEM (*n* = 8 per group). *, *P* < 0.05; **, *P* < 0.01; ***, *P* < 0.001.

To verify the validity of the sequencing data, we next randomly picked 12 differential lncRNAs (9 up-regulated lncRNAs and 3 down-regulated lncRNAs) from the dysregulated lncRNAs between the Control and PA-treated C2C12 myotubes and detected their expression levels using the qRT-PCR method (the primers were listed in Supplementary Table S1). Among the 12 lncRNAs, ENSMUST00000184420, ENSMUST00000160839, NONMMUT124495.1, NONMMUT033416.2, ENSMUST00000139662 and ENSMUST00000184126 were up-regulated while ENSMUST00000148740, ENSMUST00000170150 and NONMMUT080010.1 were down-regulated in the PA-treated C2C12 myotubes compared with the control cells via qPCR detection (all *P* < 0.05, Figure 3A–I). The qPCR results showed that the changes in lncRNA NONMMUT013870.2, NONMMUT016415.2 and NONMMUT022119.2 did not differ significantly (Figure 3J–L). The expression trends of the 12 selected lncRNAs were validated via qPCR results and were nearly consistent with the sequencing data, confirming that the lncRNA sequencing data were reliable. Thus, our results indicated that a series of lncRNAs were differentially expressed in the Control and PA-treated C2C12 myotubes and may be related to PA-induced insulin resistance. Similarly, we also chose some well-known IR-related genes from the sequencing analysis and detected their expression by qPCR. Pyruvate dehydrogenase kinase isoenzyme 4 (Pdk4) [35,36],and angiopoietin like 4 (Angptl4) [37], which were reported in promotion of IR, were observed up-regulated in the PA-treated C2C12 myotubes as shown in Supplementary Figure S2A,B. In contrary, protein kinase AMP activated gamma 3 (Prkag3) [38] and glucose transporter member 4 (Glut4) (also named Slc2a4) [39–41], whose deficiency were associated with IR, were observed with the decreased tendency in the PA-treated C2C12 myotubes as shown in Supplementary Figure S2C,D and Glut4 expression was down-regulated significantly. We further detected the expression levels of these differentially expressed lncRNAs in the skeletal muscle of *db/db* mice. Among these dysregulated lncRNAs, five of twelve lncRNAs in the *db/db* mice (ENSMUST00000184420, ENSMUST00000160839, NONMMUT124495.1, NONMMUT033416.2 and ENSMUST0000018412626) showed the consistent expression patterns to those in the PA-treated C2C12 myotubes (Figure 4). In summary, several lncRNAs that were responsive to PA stimulation *in vitro* and dysregulated in the skeletal muscle from *db/db* mouse models may participate in skeletal muscle IR.

### lncRNA–mRNA interaction network construction

**Figure 5:**
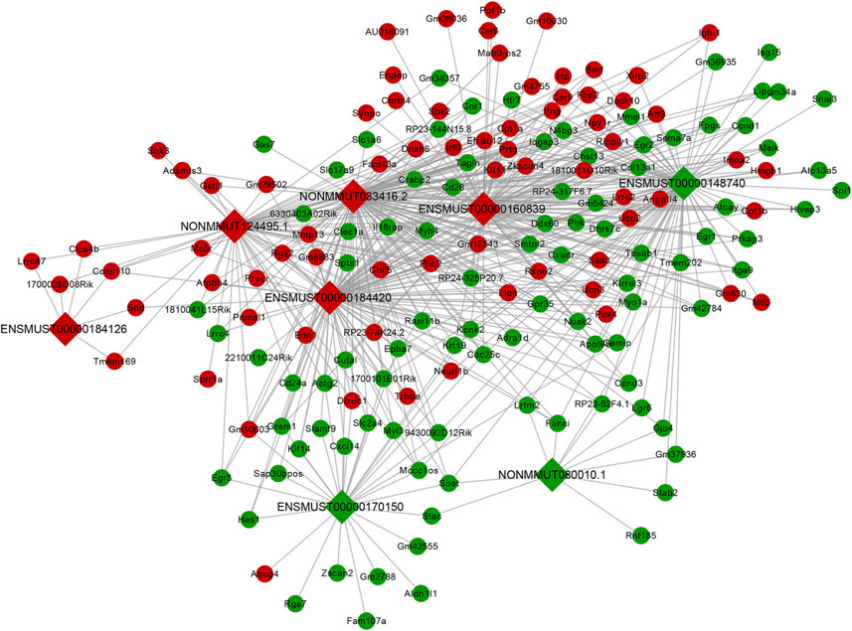
Construction of a co-expression network between the differentially expressed lncRNAs and mRNAs Circular and square nodes represent mRNAs and lncRNAs, respectively. Red represents up-regulation and green represents down-regulation. Furthermore, edges represent interactions between lncRNAs and mRNAs (*P* value< 0.05 and correlation coefficient > 0.90)

The lncRNA–mRNA co-expression networks provided us a reliable and powerful method of predicting lncRNA functions. To further examine how lncRNAs cooperated with target genes to regulate muscle function, co-expression analysis was performed on the differentially expressed lncRNAs and the corresponding differentially expressed mRNAs via the Spearman correlation analyses. After calculating the correlations between 8 lncRNAs and 217 mRNAs, 167 differentially expressed mRNAs were co-expressed with differentially expressed lncRNAs (Pearson coefficient > 0.90, *P* < 0.05) in the Supplementary Table S5. The network analysis showed that one lncRNA could correlate with many target mRNAs, implying a potential role of lncRNAs in skeletal muscle IR. LncRNA ENSMUST00000160839 was coexpressed with 80 mRNAs and ENSMUST00000148740 was coexpressed with 88 mRNAs as shown in Figure 5. This may be indicative of key lncRNA–mRNA interactions and uncover the potential regulatory roles of the candidate lncRNAs. Notably, we found that ENSMUST00000160839 was coexpressed with insulin sensitivity-related genes, including pyruvate dehydrogenase kinase isoenzyme 4 (Pdk4) (Pearson coefficient = 0.9535, *P* = 0.0032) and High-mobility group box 1 (Hmgb1) (Pearson coefficient = 0.993, *P* = 0.000078) and protein kinase AMP-activated non-catalytic subunit gamma 3 (Prkag3) (Pearson coefficient = −0.937, *P* = 0.006).

## Discussion

A detailed characterization of lncRNA on skeletal muscle glucose and lipid metabolism is essential for identifying new regulators and their proposed relevance related to insulin resistance (IR) pathophysiology and prevention. Efforts have been made to elucidate the complex lncRNAs profiles in insulin stimulation status or IR-related diseases. One study identified that more than 150 lncRNAs were dysregulated in the skeletal muscles from insulin-treated mice, which were related to glucose and lipid utilization [42]. Additionally, Huang et al. revealed that lncRNA H19 expression levels were significantly decreased in skeletal muscle tissue from T2DM subjects and high-fat-diet (HFD)-induced IR mouse models, leading to the impaired insulin signaling and decreased glucose uptake [43,44]. While several lncRNAs were described in these studies, a comprehensive view of IR-related lncRNAs in skeletal muscle cells remains unclear. In the present study, we used RNA-sequencing analysis to analyze the lncRNAs between PA-induced IR C2C12 myotubes and control ones, and tried to identify some important lncRNAs for regulating insulin sensitivity, which may provide the potential targets for treating insulin resistance and related metabolic diseases.

The present lncRNA expression profile gave us a deep comprehension of myotube-derived lncRNAs under IR status induced by PA as shown in Figure 1A,B and Supplementary Table S2. Our previous study also characterized 331 lncRNAs (|fold change| > 2, *q* < 0.05) differentially expressed in the skeletal muscle of *db/db* mice [30]. Compared with the data on mouse skeletal muscle lncRNA sequencing, fewer differential lncRNAs were detected in C2C12 myotubes and only 10 lncRNAs were overlapped between the skeletal muscle tissues and cells. As many studies have reported, lncRNAs showed tissue-specific expression patterns across various tissues with the regulatory specificity [45] and presented cell-type-specific expression patterns even in the same tissue [46]. Satellite cells [47], adult stem cells [47], macrophages [48], and vascular endothelial cells [49] reside in the skeletal muscle that possesses a three-dimensional microenvironment. Additionally, skeletal muscle fibers can be classified as fast oxidative-glycolytic, fast glycolytic, slow oxidative as well as various hybrid muscle fibers based on myosin heavy-chain (MHC) isoforms [50]. Different skeletal muscle fibers possess different metabolic properties [50]. The relatively low overlapping may also result from the complex composition of skeletal muscle. Thus, using the C2C12 myotubes model may help identify potential lncRNA regulators under IR status.

Based on functional enrichment analysis of differentially regulated mRNAs, these GO terms as shown in Figure 2A were in line with previous reports that the impaired fatty acid oxidation or lipid oxidation capacity could induce ectopic lipid accumulation in skeletal muscle cells [51] or tissues [52,53], which strongly accelerates IR. KEGG pathway analysis predicted the differential lncRNAs that were predicted to be correlated with “fatty acid degradation”, “fatty acid metabolism” and “regulation of lipolysis in adipocytes” in Figure 2B, which were involved in lipid metabolism and were consistent with these enriched GO terms. In addition, various studies have demonstrated that abnormal PPAR expression was observed in skeletal muscles from IR models [54] and in T2DM [55]. PPARdelta, one kind of PPAR family, functioned as activators in lipid metabolism [56,57]. Our pathways analysis also confirmed the potential function of these dysregulated lncRNAs in “PPAR signaling pathway”. Therefore, we speculated that there may exist potential relationships among the altered lncRNA expression, dysregulation of PPAR and insulin signaling pathways in the lipid-related IR. In general, the above results gave us several hints to identify candidate lncRNAs for further characterization. Specifically, the results verified by qPCR from our PA-treated cell models showed some difference from those in diabetic *db/db* mice models as shown in Figures 3 and 4. Such a distinction is probably associated with the complex composition in skeletal muscle tissues. Considering the heterogeneity between cell line and tissues, further studies are needed to combine the application of primary myoblasts isolation experiments to confirm the expression of candidate lncRNAs and elucidate their potential role in regulating insulin sensitivity.

LncRNA–mRNA co-expression network analysis helped uncover the potential regulatory roles of candidate lncRNA ENSMUST00000160839 as shown in Figure 5. Among the associated genes in Supplementary Table S5, Pdk4 (Pearson coefficient = 0.9535, *P* = 0.0032) and Hmgb1 (Pearson coefficiency = 0.993, *P* = 0.000078) were both positively related correlated with expression of lncRNA ENSMUST00000160839, while Prkag3 and lncRNA ENSMUST00000160839 were markedly negatively correlated (Pearson coefficiency = −0.937, *P* = 0.006). Pdk4, which regulates pyruvate dehydrogenase complex (PDC) activity, is considered as a vital regulator of metabolic fuel switching within the skeletal muscle [58]. D. Cameron-Smith et al. observed the induced expression levels of Pdk4 by free fatty acids (FFA) stimulation and Pdk4 up-regulation in myotubes cultured from obese and T2DM patients [36]. Additionally, Pdk4-knockout mice presented an improved metabolic phenotype and were protected against diet-induced IR in their skeletal muscle [35]. Another IR-related gene Hmgb1, is an important mediator during diabetes onset and progression [59]. Increasing evidence also indicated its importance in inflammation, which led to the skeletal muscle dysfunction [60]. As a subunit of 5-AMP-activated protein kinase (AMPK) [61], Prkag3 played a key role in lipid metabolism of skeletal muscle and its decreased expression failed to induce glucose uptake in skeletal muscle stimulated by an AMPK activator [38]. Notably, Pdk4 expression was confirmed up-regulated in PA-treated myotubes by qPCR results as shown in Supplementary Figure S2 and this trend was according to previous reports, which may give hints of a important lncRNA–mRNA pair (ENSMUST00000160839-Pdk4). This analysis was conducted to provide functional implications of the candidate lncRNA ENSMUST00000160839 by analyzing its association with mRNAs. Although three biological replicates were used in co-expression analysis in previous studies [62–65], the increased sample size in the present study will help validate the results.

Till now, the reported lncRNAs involved in skeletal muscle metabolism play various roles in insulin substrate receptors (INSR) expression [66], the autophagy pathway [67] and AMPK activity [68]. Similarly, the diverse mechanisms for modulating insulin sensitivity in skeletal muscle cells enable exploring how this candidate lncRNA regulated these genes from the co-expression network. Consistent with their predominantly in cytoplasmic localization, muscle-derived lncRNAs have been increasingly described as miRNA sponges with targeting downstream genes [69,70]. For example, muscle-enriched lncRNA H19 acted to enhance muscle insulin sensitivity like a sponge by sequestering let-7 targeting dual specificity phosphatase 27 (DUSP27) expression levels [68]. Thus, in the subsequent studies, we will explore and validate whether molecular factors involved in the co-expression network analysis that preferentially regulate insulin sensitivities in skeletal muscles are mediated by the candidate lncRNA ENSMUST00000160839.

In conclusion, the present study profiled the differential expression of lncRNAs between PA-treated and control C2C12 myotubes and uncovered the potential regulatory roles of candidate lncRNA ENSMUST00000160839. Our study extended the skeletal muscle lncRNA database and provided novel potential regulators for future genetic and molecular studies on insulin resistance, which may help for prevention and treatment of the related metabolic diseases. However, further exploration and validation of lncRNAs expression in primary myoblasts need to be done. Additionally, the detailed biological effects of these lncRNAs on skeletal muscle insulin resistance remained to be elucidated in the future study.

## Supplementary Material

Supplementary Figures S1-S2

Supplementary Tables S1-S6

## Abbreviations

GO: Gene Ontology
IR: insulin resistance
KEGG: Kyoto encyclopedia of genes and genomes
lncRNA: long noncoding RNA
T2DM: Type 2 diabetes mellitus
