# Supplementary figures and images for: RNA-sequencing analysis reveals the potential contribution of lncRNAs in palmitic acid-induced insulin resistance of skeletal muscle cells
Source: Biosci Rep. 2020 Jan 2;40(1):BSR20192523. doi: 10.1042/BSR20192523 (PMC6944669; doi:10.1042/BSR20192523)

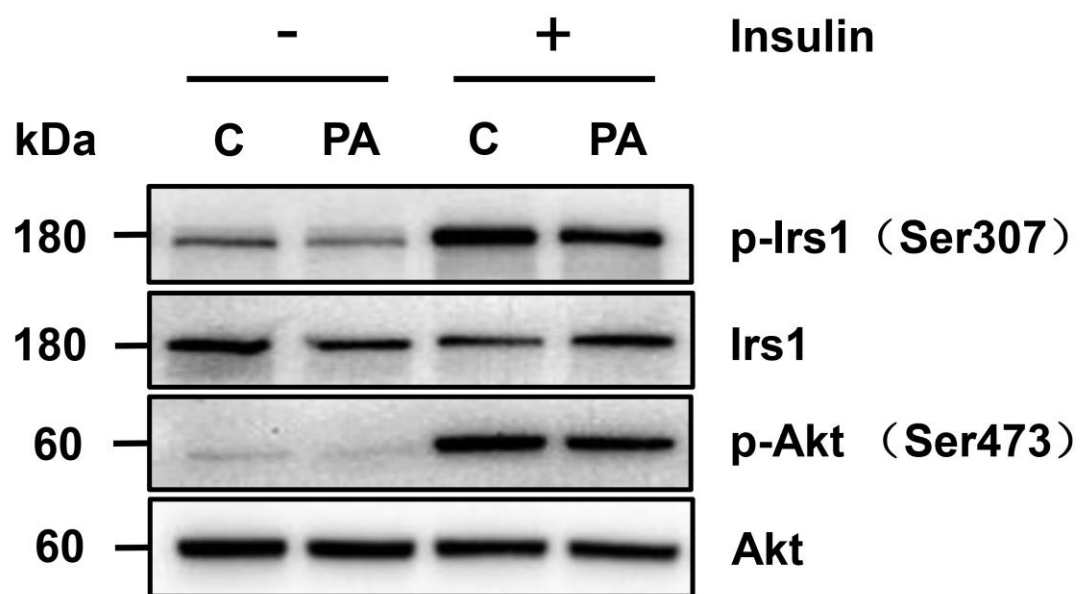

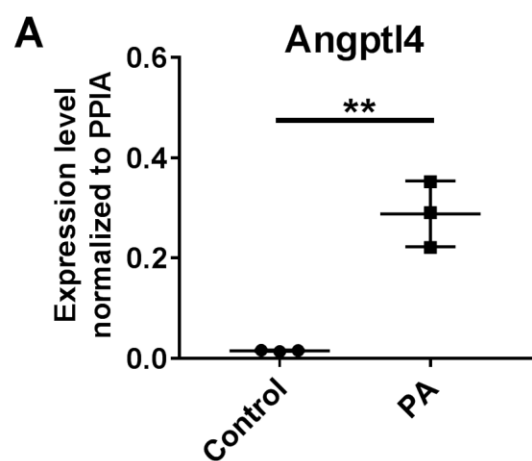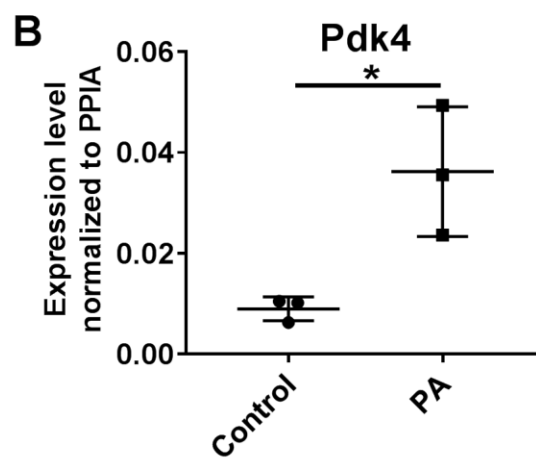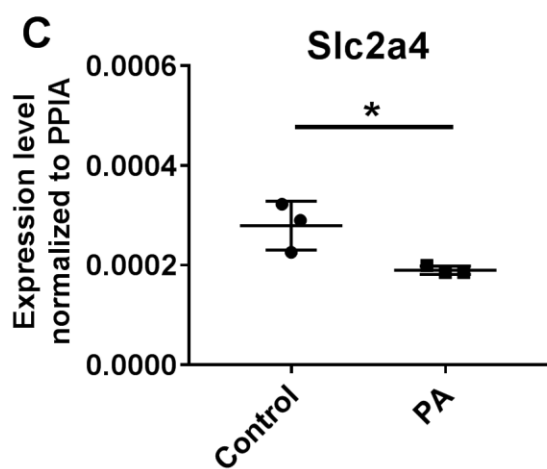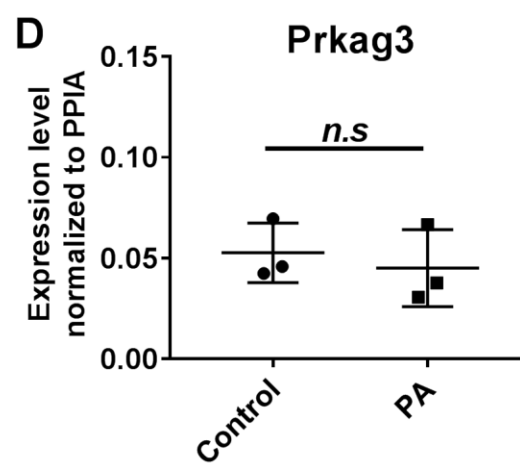

Supplement: Supplementary Figures S1-S2 [file BSR-2019-2523_supp.pdf]
